# Design and analysis of an interleaved step-up DC–DC converter with enhanced characteristics

**DOI:** 10.1038/s41598-024-65171-5

**Published:** 2024-06-22

**Authors:** Majid Hosseinpour, Elham Seifi, Ali Seifi, Mahdi Shahparasti

**Affiliations:** 1 Department of Electrical Engineering, University of Mohaghegh Ardabili, Ardabil, Iran; 2 School of Technology and Innovations, University of Vaasa, Vaasa, Finland

**Keywords:** Interleaved converter, Improved voltage gain, Voltage rectifier, Input current ripple, Energy science and technology, Electrical and electronic engineering

## Abstract

In this paper, an interleaved DC–DC step-up converter with improved characteristics based on a voltage multiplier rectifier is presented. The proposed converter is presented and analyzed for two different operating duty regions including operating region 1 (0 < *D* ≤ 0.5), and operating region 2 (0.5 ≤ *D* < 1). This converter can be used in various applications such as energy storage, electric vehicles, and renewable energy systems. This converter is composed of two stages: an interleaved boost stage and a voltage multiplier rectifier stage, which collectively forms its general structure. The interleaved boost stage is a type of two-phase boost converter that transforms the input DC voltage into a high-frequency AC square waveform. This waveform can be readily filtered using smaller capacitors. The square-shaped voltage waveform from the interleaved boost stage is rectified and converted to a high DC voltage by the Voltage Multiplier Rectifier (VMR) stage. The operating regions, the evaluation of the steady-state condition, the voltage gain of the proposed converter's parasitic and ideal models as well as its losses and efficiency analysis have been evaluated. The proposed converter has an efficiency of 97% at the output power of 150 W. The proposed converter is simulated to convert a voltage of 25–159.5 V and to validate the mathematical relationships and simulation results, a laboratory prototype has been developed. The simulation and experimental results show the precision of the performance of the proposed interleaved boost converter.

## Introduction

In recent years, one of the considerable problems is global warming and its environmental effects. The pollution produced by fossil fuels such as gas and oil is the most significant reason for these problems. Thus, there has been an increased focus on renewable energy resources such as solar energy, fuel cells, and wind power. Renewable energy sources, including fuel cells and photovoltaic arrays, produce low amplitude output voltages. As a result, the need for power electronics converters is felt to increase their voltage and bring it to the appropriate range for use by the load or connecting to the network^1^. For this purpose, DC–DC converters with high voltage gain are a suitable choice^2,3^.

In the conventional step-up DC–DC converter, the switch voltage stress is substantial and is equivalent to the output voltage^4^. Therefore, the traditional step-up converter needs switches with higher ratings to withstand higher voltage stress. Furthermore, choosing higher duty cycles is necessary to attain high voltage gain, which results in diode reverse recovery issues, conduction losses, and voltage spikes^5^. Depending on the requirements of the application, a variety of DC–DC converters with high voltage gain capability are now available. Isolated and non-isolated converters are the two main types of DC–DC converters with high voltage gain. Isolated converters have higher hardware costs and larger volumes. They also have to deal with significant obstacles such as the thermal effect, high price, leakage inductance, core saturation, and high voltage stress on power switches^6,7^. Non-isolated DC–DC converters with high voltage gain are considered due to their boost capability, high efficiency, and proper voltage regulation. These converters are widely employed in many different applications, including DC nano grids^8^, electric vehicles^9^, photovoltaic (PV) systems^10^, and fuel cells (FC)^11^. The primary types of non-isolated DC–DC converters are cascaded converters, coupled inductor converters, switched capacitor converters, and interleaved converters.

The interleaved technique is an internal connection of multiple switching cells that increases the effective pulse frequency by synchronizing several smaller sources and operating them with a suitable phase shift. In medium/high power applications, the interleaved structure can be used to reduce input current ripple, enhance dynamic response, reduce magnetic component sizes, and improve thermal distribution. The interleaved structure decreases input current ripple and increases converter ripple frequency without increasing switching losses or switching device stresses. Therefore, it can reduce the need for filtering and energy storage, thereby significantly improving the power conversion density without compromising the efficiency of the converter. The popularity of interleaved boost DC–DC converters in applications such as energy storage^12^, electric vehicles^13^, and renewable energy systems^14^ can be attributed to these advantages.

Various structures have been proposed for interleaved converters. A DC–DC converter utilizing a modified triple-boosting architecture (MTB) interleaved with modified switched inductor capacitors (MSIC) is presented in^15^ to attain high voltage gain in photovoltaic applications. A large number of devices and a relatively high switching frequency result in high losses, which are the problems with this structure. An n-phase Interleaved Complementary Current-fed Topology (n-phase ICCFT) with two pulse-width-modulation (PWM) schemes is introduced in^16^ to implement the interleaving of the n-phases. This converter has three power switches, which results in a considerable switching loss for this structure and its voltage gain is not a value corresponding to the structure containing three power switches. An interleaved step-up DC–DC converter for high-voltage applications based on a quasi-Z-source is discussed in^17^. All power devices in this converter employ hard switching, which is unsuitable for increasing the switching frequency and reducing switching losses. In^18^, another type of multiphase interleaved step-up converter with soft switching and using an additional resonant circuit is presented. This converter works at a specific frequency which results in low power density. Also, high voltage gain is not properly met in high-duty cycles. The interleaved multilevel boost converter is described in^19^ for high-voltage DC microgrid applications. As a result of the increased number of components and voltage gain achieved through the use of multiple stages in this converter, intricate power and control circuits have been developed. In^20^, an interleaved structure along with the multiplier cell is introduced to boost the voltage gain but, because there are so many components in the converter, its efficiency is not impressive. The interleaved boost converter presented in^21^ can operate in different regions with different duty cycle values. However, the maximum voltage gain is limited and does not work properly at high powers. In^22^, a symmetrical three-winding coupled inductor (TW-CI) based interleaved step-up converter is introduced. The main issue related to leakage inductance is the occurrence of voltage spikes in the MOSFET switch, leading to considerable power loss and potentially damaging the power switch. An interleaved Luo converter, which combines the advantages of both switched capacitors and interleaved topologies, is utilized in^23^. Also, an Interleaved High-Gain Modified SEPIC (IHGM-SEPIC) DC-DC converter has been presented in^24^ for PV applications. In both of these structures, the number of elements used, particularly in^24^, is crucial because it impacts the volume and efficiency of the converter.

The converter’s capability for high voltage and high power applications is increased when voltage multipliers (VM) are used in interleaved converters^25–32^. An interleaved step-up converter with a bi-fold Dickson voltage multiplier is reported in^25^ and can be used to link medium-voltage distribution buses with low-voltage renewable energy sources. To obtain high voltage gain, this converter employs a switched capacitor technique. Large instantaneous currents passing through the capacitors cause additional power losses, electromagnetic noises, and switch current stress, which is the fundamental drawback of converters based on switched capacitors. Also, the limited range of duty cycle values and a large number of elements are limitations of this structure. The converter described in^26^ has a modified Dickson Charge Pump Voltage Multiplier on the output side and a two-phase interleaved step-up converter on the input side which leads to reduced magnetic storage requirements and smoother input current. To rectify or regulate the output voltage, the converter requires an LC filter or output diode. Also, this converter has lower efficiency. This converter needs a capacitor with a high nominal voltage at the output and does not offer a way to lessen the voltage stress on the diode. An interleaved DC–DC step-up converter with an input-parallel output series is introduced in^27^. This converter has two duty regions with different input current ripples and the same voltage gain. However, it has a reverse polarity output. Only in high-duty cycles, this converter can produce a high voltage gain; in lower-duty cycles, the voltage gain is significantly decreased.

An interleaved boost converter with a fixed frequency sliding mode control strategy is provided in^28^ to guarantee the system's steady operation. Relatively low voltage gain and complexity in controller design are disadvantages of this converter. In addition, it requires additional current sensors, which increases the hardware cost as a result. In^29^, a tri-state interleaved boost converter is presented. This converter uses a diode and an extra switch to provide a freewheeling time, which keeps the output capacitor's charging time fixed. The dynamic response is enhanced by the tri-state interleaved converter, but the circuit has the same gain as a traditional boost converter and requires more complex control. A multi-input step-up DC–DC converter that is based on the Cockcroft-Walton (CW) multiplier is shown in^30^. In this converter, the current of the input inductors must be controlled by a current balancer. The power density is further limited by the large volume of the required capacitors. Every capacitor in the Cockcroft-Walton voltage multiplier cell experiences the same voltage stress. Increasing the number of the stages of multiplier cells leads to an increase in the output impedance of this cell. Consequently, the converter’s efficiency declines. A Greinacher voltage multiplier-based interleaved boost converter is described in^31^. This converter operates properly just for limited duty cycle values. In^32^, an active clamp circuit and a voltage multiplier (VM) are combined for high-voltage applications to introduce and investigate a family of interleaved current-fed step-up DC–DC converters. Because a large number of power devices are used in this converter, the efficiency of the converter is affected by this issue.

In this paper, an interleaved DC–DC step-up converter with improved characteristics such as higher efficiency and relatively higher real voltage gain based on a voltage multiplier rectifier is presented. The power switches experience significantly less voltage stress compared to the output voltage. To reduce conduction losses, power switches with lower on-state resistances are employed. The interleaved structure significantly reduces the input current ripple, extending the useful life of renewable energy systems.

This article is structured as follows: section “Proposed converter and operation principles” provides an overview of the structure and operation principles of the proposed converter in continuous conduction mode (CCM). Section “Steady-state analysis” presents the steady-state analysis of the proposed converter, which includes the calculation of the ideal and actual gain coefficient, the design of parameters in different operational areas, and finally, the loss and efficiency of the proposed converter. Section “Steady-state analysis” presents the steady-state analysis of the proposed converter, which includes determining the ideal and real voltage gain, designing the parameters in various operating regions, and calculating the converter's efficiency and loss. In section “Comparison of interleaved converters”, the proposed converter is compared to similar converters to analyze the converter’s feasibility. Section “Simulation and experimental results” also provides a detailed discussion of the simulation and laboratory results. In section “Conclusion”, the paper is finally ended.

## Proposed converter and operation principles

**Figure 1:**
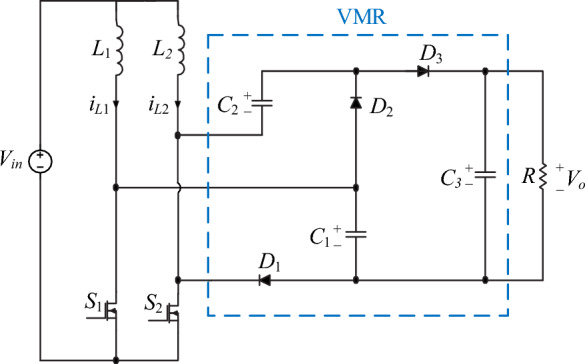
Proposed converter.

In this part, the steady-state analysis and operation principles of the proposed converter are explained. The proposed converter is shown in Fig. 1. To analyze the converter, the elements are thought to be ideal, and the capacitor’s values are thought to be large enough to overlook voltage ripple. An interleaved step-up stage and a voltage multiplier rectifier (VMR) make up the general structure of the proposed converter. The proposed converter includes two power MOSFETs *S*_1_, *S*_2_, two inductors *L*_1_, *L*_2_, three capacitors *C*_1_, *C*_2_, *C*_3_, and three diodes *D*_1_, *D*_2_, *D*_3_. In the continuous conduction mode (CCM), the proposed converter has been analyzed. A switching logic has been developed for the operation of the proposed converter in two operating regions including operating region 1 (0 < *D* ≤ 0.5) and operating region 2 (0.5 ≤ *D* < 1) which leads to different voltage gains. *D* is the proposed converter's duty cycle, or the ON time of switches to the switching period. The following subsections provide a comprehensive analysis of the converter's mathematical modeling.

### Operating Region 1 (0 < *D* ≤ 0.5)

**Figure 2:**
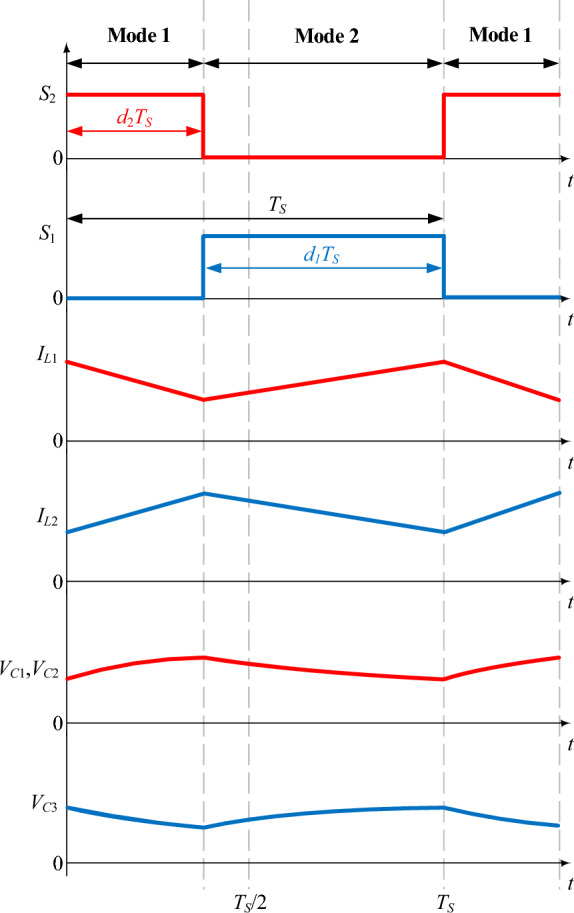
Essential operating waveforms of the proposed converter for 0 < *D* ≤ 0.5.

Figure 2 displays the fundamental operating waveforms of the proposed converter for region 1 (0 < *D* ≤ 0.5). In this region, *S*_1_ and *S*_2_ are switched in a complementary manner. The proposed converter includes two switching modes (mode 1 and mode 2) during the switching period (*T*_*S*_), as shown in Fig. 2.

#### Mode 1 (*S*_1_ off and *S*_2_ on)

**Figure 3:**
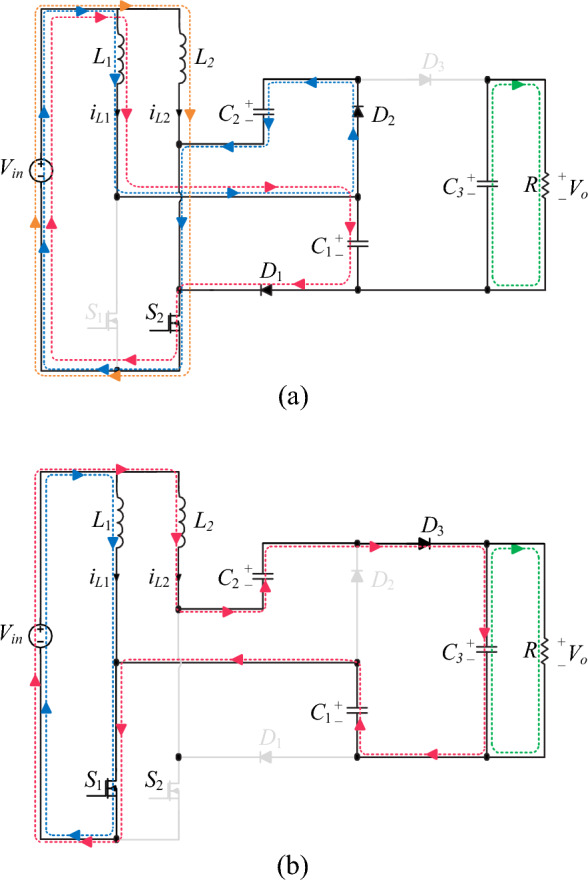
Equivalent circuit of different switching modes of the proposed converter in operating region 1: (**a**) *S*_1_ off and *S*_2_ on (**b**) *S*_1_ on and *S*_2_ off.

The equivalent circuit of the proposed converter when *S*_1_ is off and *S*_2_ is on is shown in Fig. 3a. In this mode, diodes *D*_1_ and *D*_2_ are forward-biased and diode *D*_3_ is reverse-biased. Inductor *L*_1_ discharges its energy into capacitors *C*_1_ and *C*_2_. Therefore, its current slope is negative according to Fig. 2. The inductor *L*_2_ is charged through the input voltage *V*_*in*_. In Fig. 2, the current slope of inductor *L*_2_ is positive. The energy stored in the capacitor *C*_3_ is discharged into the output load *R*. The resulting equations in mode 1 are according to relations (1), (2).

1 $$\left\{ {\begin{array}{*{20}l} {L_{1} \frac{{di_{L1} }}{dt} = v_{L1} = V_{in} - V_{C2} } \hfill \\ {L_{2} \frac{{di_{L2} }}{dt} = v_{L2} = V_{in} } \hfill \\ \end{array} } \right.$$

2 $$\left\{ {\begin{array}{*{20}l} {i_{C1} = i_{C2} = \frac{{I_{in} - i_{L2} }}{2}} \hfill \\ {i_{C3} = - i_{o} } \hfill \\ \end{array} } \right.$$

#### Mode 2 (*S*_1_ on and *S*_2_ off)

The equivalent circuit of the proposed converter when *S*_1_ is on and *S*_2_ is off is shown in Fig. 3b. In this mode, diodes *D*_1_ and *D*_2_ are reverse biased and diode *D*_3_ is forward biased. The stored energy is delivered to the output load and output capacitor *C*_3_ by inductor *L*_2_ and capacitors *C*_1_ and *C*_2_. Therefore, according to Fig. 2, the current slope of the inductor *L*_2_ and the voltage slope of the capacitors *C*_1_ and *C*_2_ are negative in this mode. The inductor *L*_1_ is charged through the input voltage *V*_*in*_. Also, according to the positive slope of the inductor *L*_1_'s current in Fig. 2, it can be concluded that the inductor *L*_1_ is charging. The resulting equations in mode 2 are according to relations (3), (4).

3 $$\left\{ {\begin{array}{*{20}l} {L_{1} \frac{{di_{L1} }}{dt} = v_{L1} = V_{in} } \hfill \\ {L_{2} \frac{{di_{L2} }}{dt} = v_{L2} = V_{in} + V_{C1} + V_{C2} - V_{C3} } \hfill \\ \end{array} } \right.$$

4 $$\left\{ {\begin{array}{*{20}l} {i_{C1} = i_{C2} = i_{L1} - I_{in} = - i_{L2} } \hfill \\ {i_{C3} = i_{L2} - i_{o} } \hfill \\ \end{array} } \right.$$

### Operating region 2 (0.5 ≤ *D* < 1)

**Figure 4:**
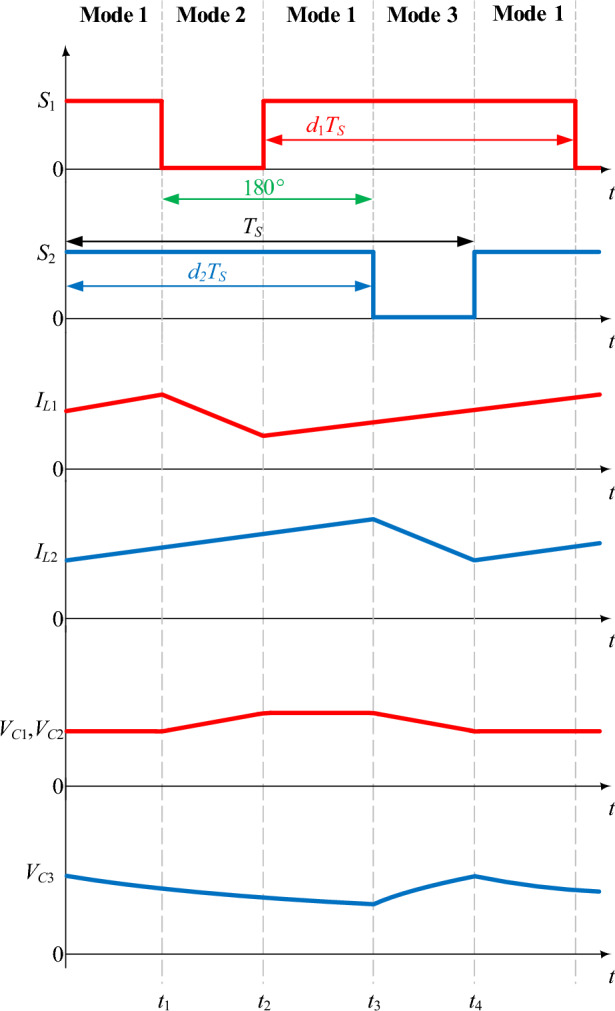
Operating waveforms of the proposed converter for 0.5 ≤ *D* < 1.

The essential operating waveforms of the proposed converter for region 2 (0.5 ≤ *D* < 1) are shown in Fig. 4. In this region, there is a 180^°^ phase difference between *S*_1_ and *S*_2_. From Fig. 4, it can be seen that the proposed converter has three switching modes (mode 1, mode 2, and mode 3) in the switching period (*T*_*S*_).

#### Mode 1 (*S*_1_ on and *S*_2_ on)

**Figure 5:**
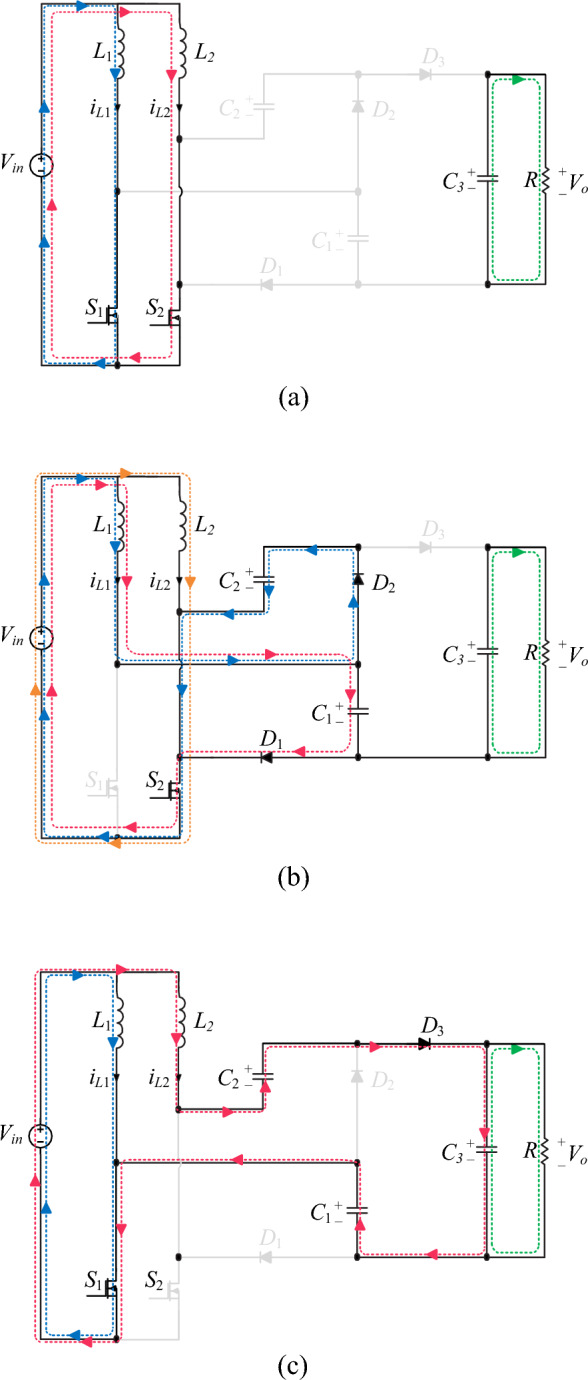
Equivalent circuit of different switching modes of the proposed converter in operating region 2: (**a**) *S*_1_ on and *S*_2_ on (**b**) *S*_1_ off and *S*_2_ on (**c**) *S*_1_ on and *S*_2_ off.

The equivalent circuit of the proposed converter when *S*_1_ and *S*_2_ are on is shown in Fig. 5a. In this mode, the inductors are charged through the input source, and the diodes are reverse-biased. The energy stored in the capacitor *C*_3_ is discharged into the output load *R*. Capacitors *C*_1_, *C*_2_, and *C*_3_ is neither charged nor discharged. As seen in Fig. 4, the inductors’ current slope is positive, indicating that they are charged in this mode. Also, the voltage slope of capacitors *C*_1_, *C*_2_, and *C*_3_ remains constant. The resulting equations in mode 1 are in the form of relations (5), (6).

5 $$\left\{ {\begin{array}{*{20}l} {L_{1} \frac{{di_{L1} }}{dt} = v_{L1} = V_{in} } \hfill \\ {L_{2} \frac{{di_{L2} }}{dt} = v_{L2} = V_{in} } \hfill \\ \end{array} } \right.$$

6 $$\left\{ {\begin{array}{*{20}l} {i_{L1} + i_{L2} = I_{in} } \hfill \\ {i_{C3} = - i_{o} } \hfill \\ \end{array} } \right.$$

#### Mode 2 (*S*_1_ off and *S*_2_ on)

The equivalent circuit of the proposed converter when *S*_1_ is off and *S*_2_ is on is shown in Fig. 5b. In this mode, diodes *D*_1_ and *D*_2_ are forward-biased biased, and diode *D*_3_ is reverse-biased. *L*_1_ discharges its energy into capacitors *C*_1_ and *C*_2_. Therefore, its current slope is negative according to Fig. 4. The inductor *L*_2_ is charged through the input voltage *V*_*in*_. In Fig. 4, the current slope of inductor *L*_2_ is positive. The energy stored in the capacitor *C*_3_ is discharged into the output load *R*. The resulting equations in mode 1 are according to relations (7), (8).

7 $$\left\{ {\begin{array}{*{20}l} {L_{1} \frac{{di_{L1} }}{dt} = v_{L1} = V_{in} - V_{C1} = V_{in} - V_{C2} } \hfill \\ {L_{2} \frac{{di_{L2} }}{dt} = v_{L2} = V_{in} } \hfill \\ \end{array} } \right.$$

8 $$\left\{ {\begin{array}{*{20}l} {i_{C1} = i_{C2} = \frac{{I_{in} - i_{L2} }}{2}} \hfill \\ {i_{C3} = - i_{o} } \hfill \\ \end{array} } \right.$$

#### Mode 3 (*S*_1_ on and *S*_2_ off)

The equivalent circuit of the proposed converter when *S*_1_ is on and *S*_2_ is off is shown in Fig. 5c. In this mode, diodes *D*_1_ and *D*_2_ are reverse biased and diode *D*_3_ is forward biased. The stored energy is delivered to the output load and output capacitor *C*_3_ by inductor *L*_2_ and capacitors *C*_1_ and *C*_2_. Therefore, according to Fig. 4, the current slope of the inductor *L*_2_ and the voltage slope of the capacitors *C*_1_ and *C*_2_ are negative in this mode. The inductor *L*_1_ is charged through the input voltage *V*_*in*_. Also, according to the positive slope of the inductor *L*_1_'s current in Fig. 4, it can be concluded that the inductor *L*_1_ is charging. The resulting equations in mode 3 are according to relations (9), (10).

9 $$\left\{ {\begin{array}{*{20}l} {L_{1} \frac{{di_{L1} }}{dt} = v_{L1} = V_{in} } \hfill \\ {L_{2} \frac{{di_{L2} }}{dt} = v_{L2} = V_{in} + V_{C1} + V_{C2} - V_{C3} } \hfill \\ \end{array} } \right.$$

10 $$\left\{ {\begin{array}{*{20}l} {i_{C1} = i_{C2} = i_{L1} - I_{in} = - i_{L2} } \hfill \\ {i_{C3} = i_{L2} - i_{o} } \hfill \\ \end{array} } \right.$$

## Steady-state analysis

This part includes a detailed analysis of the proposed converter's ideal and real voltage gains, parameters design, voltage stress on the diodes and switches, and, lastly, the calculations of losses and efficiency in the continuous conduction mode (CCM). Since the proposed converter has two types of switching logic for duty cycles less than and greater than 0.5, the ideal and real voltage gain, as well as the design of the parameters for the two operating regions, are presented.

### Ideal voltage gain in operating region 1 (0 < *D* ≤ 0.5)

By applying the volt-second balance principle on the inductors in a switching period in the operation region 1 according to Eqs. (11) and (12), the voltage of the capacitors is obtained according to Eq. (13), which can be used to achieve the ideal voltage gain.

11 $$\left\langle {v_{L1} } \right\rangle = \int_{0}^{{DT_{S} }} {\left( {V_{in} - V_{C2} } \right)dt} + \int_{{DT_{S} }}^{{T_{S} }} {V_{in} } dt = 0$$

12 $$\left\langle {v_{L2} } \right\rangle = \int_{0}^{{DT_{S} }} {V_{in} dt} + \int_{{DT_{S} }}^{{T_{S} }} {\left( {V_{in} + V_{C2} + V_{C1} - V_{o} } \right)} dt = 0$$

13 $$V_{C1} = V_{C2} = \frac{{V_{in} }}{D}$$

Therefore, by substituting Eq. (13) into Eqs. (11) and (12), the ideal voltage gain of the proposed converter is obtained as follows.

14 $$M = \frac{{V_{o} }}{{V_{in} }} = \frac{{\left( {2 - D} \right)}}{{D\left( {1 - D} \right)}}$$

As it is known, the voltage of capacitor *C*_1_ is equal to the voltage of capacitor *C*_2_. It is also worth mentioning that the ideal voltage gain calculated for the duty cycle is valid in the range (0 < *D* ≤ 0.5).

### Ideal voltage gain in operating region 2 (0.5 ≤ *D* < 1)

By applying the volt-second balance principle on the inductors in a switching period in the operation region 2 according to Eqs. (15) and (16), the voltage of the capacitors is obtained according to Eq. (17), and by using these equations, the ideal voltage gain of the proposed converter can be obtained.

15 $$\left\langle {v_{L1} } \right\rangle = \int_{0}^{{DT_{S} }} {V_{in} dt} + \int_{{DT_{S} }}^{{T_{S} }} {\left( {V_{in} - V_{C2} } \right)} dt = 0$$

16 $$\left\langle {v_{L2} } \right\rangle = \int_{0}^{{DT_{S} }} {V_{in} dt} + \int_{{DT_{S} }}^{{T_{S} }} {\left( {V_{in} + V_{C2} + V_{C1} - V_{C3} } \right)} dt = 0$$

17 $$V_{C1} = V_{C2} = \frac{{V_{in} }}{{\left( {1 - D} \right)}}$$

Therefore, by substituting Eq. (17) into Eqs. (15) and (16), the ideal voltage gain of the proposed converter is obtained as follows.

18 $$M = \frac{{V_{o} }}{{V_{in} }} = \frac{3}{{\left( {1 - D} \right)}}$$

As it is known, the voltage of capacitor *C*_1_ is equal to the voltage of capacitor *C*_2_. It is also worth mentioning that the ideal voltage gain calculated for the duty cycle is valid in the range (0.5 ≤ *D* < 1).

### Real voltage gain

**Figure 6:**
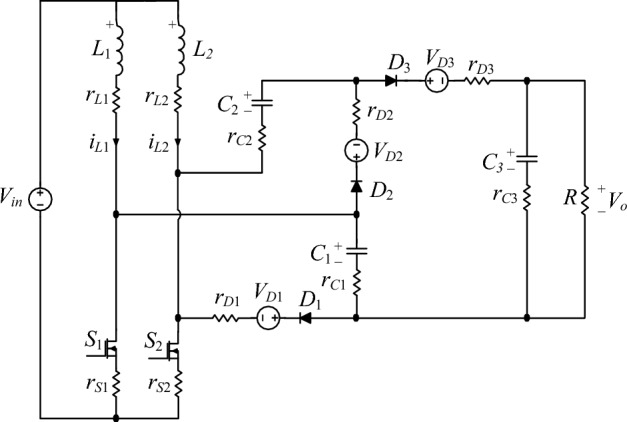
Effect of parasitic elements in the proposed converter’s structure.

This section presents an analysis of the impact of parasitic elements on the output voltage and efficiency. Figure 6 illustrates the proposed converter circuit with parasitic elements. The inductors' equivalent series resistance (ESR) includes *r*_*L*1_ and *r*_*L*2_. *r*_*S*1_, *r*_*S*2_ represent the switches' on-state resistances, while *r*_*D*1_, *r*_*D*2_ and *r*_*D*3_ are the internal resistances of the diodes and *V*_*D*1_, *V*_*D*2_ and *V*_*D*3_ are their forward bias voltage drops. *r*_*C*1_, *r*_*C*2_ and *r*_*C*3_ are the equivalent series resistance (ESR) of capacitors *C*_1_, *C*_2_ and *C*_3_.

The operation principles of the proposed converter in real conditions are similar to the operation principles in the ideal state, and the parasitic elements are also considered in the equivalent circuit and the governing equations of the circuit. Similar to ideal voltage gain calculations, by applying the volt-second balance principle to the inductors and taking into account the parasitic parameters, the real voltage gain of the proposed converter is calculated in operating region 1 (0 < *D* ≤ 0.5) according to Eq. (19) and in operating region 2 (0.5 ≤ *D* < 1) according to Eq. (20).

19 $$\begin{gathered} M = \frac{{V_{o} }}{{V_{in} }} = \frac{{\left( {\frac{{\left( {2 - D} \right)}}{{D\left( {1 - D} \right)}}} \right) - \left( {\frac{{2V_{D2} }}{{V_{in} }}} \right) - \left( {\frac{{V_{D3} }}{{V_{in} }}} \right)}}{{1 + \left[ {ar_{S2} + br_{L2} + cr_{D2} + dr_{C2} + er_{L1} + fr_{S1} + gr_{D3} + gr_{C1} } \right]h\frac{1}{{R_{o} }}}} \hfill \\ \left\{ {\begin{array}{*{20}l} {a = \frac{{\left( {D^{2} - 4D + 4} \right)}}{{\left( {1 - D} \right)}}} \hfill & {b = \frac{D}{{\left( {1 - D} \right)}}} \hfill \\ {c = \frac{{\left( {2D^{2} - 4D + 2} \right)}}{{\left( {1 - D} \right)}}} \hfill & {d = \frac{{\left( {D^{2} - D - 2} \right)}}{{\left( {1 - D} \right)}}} \hfill \\ {e = \frac{{\left( {4 - 4D} \right)}}{D}} \hfill & {f = \frac{{\left( { - D^{3} + 5D^{2} - 8D + 4} \right)}}{{D\left( {1 - D} \right)}}} \hfill \\ {g = D} \hfill & {h = \frac{1}{{D\left( {1 - D} \right)}}} \hfill \\ \end{array} } \right. \hfill \\ \end{gathered}$$

20 $$\begin{gathered} M = \frac{{V_{o} }}{{V_{in} }} = \frac{{\left( {\frac{3}{1 - D}} \right) - \left( {\frac{{2V_{D2} }}{{V_{in} }}} \right) - \left( {\frac{{V_{D3} }}{{V_{in} }}} \right)}}{{1 + \left[ {ar_{S2} + br_{L2} + cr_{S1} + dr_{L1} + 2r_{D2} + 3r_{C2} + r_{D3} + r_{C1} } \right]b\frac{1}{{R_{o} }}}} \hfill \\ \left\{ {\begin{array}{*{20}l} {a = \frac{{\left( {8 - 7D} \right)}}{{\left( {1 - D} \right)}}} \hfill & {b = \frac{1}{{\left( {1 - D} \right)}}} \hfill \\ {c = \frac{{3\left( {1 + D} \right)}}{{\left( {1 - D} \right)}}} \hfill & {d = \frac{4}{{\left( {1 - D} \right)}}} \hfill \\ \end{array} } \right. \hfill \\ \end{gathered}$$

In the following, the process of parameter design for the proposed converter has been examined. The parameter design has been checked once for the operating region 1, which has a duty cycle of less than 0.5, and repeated for the operating region 2, which has a duty cycle of more than 0.5.

### Parameter design in operating region 1 (0 < *D* ≤ 0.5)

#### Inductor selection

When the switches *S*_1_ and *S*_2_ are on, the inductors *L*_1_ and *L*_2_ have a positive current slope, and the voltage of capacitors and relations (1) and (3) can be used to determine the current ripple of inductors, which yields relations (21) and (22).

21 $$\Delta i_{L1} = \frac{{V_{in} \left( {1 - D} \right)}}{{L_{1} f_{s} }}$$

22 $$\Delta i_{L2} = \frac{{V_{in} D}}{{L_{2} f_{s} }}$$

Hence, the values of inductors *L*_1_ and *L*_2_ operating under continuous conduction mode (CCM) are obtained according to relations (23) and (24).

23 $$L_{1} = \frac{{V_{in} \left( {1 - D} \right)}}{{\Delta i_{L1} f_{s} }}$$

24 $$L_{2} = \frac{{V_{in} D}}{{\Delta i_{L2} f_{s} }}$$

The average current passing through inductors *L*_1_ and *L*_2_ is as follows:

25 $$I_{L1,avg} = \frac{{2I_{in} \left( {1 - D} \right)}}{{\left( {2 - D} \right)}}$$

26 $$I_{L2,avg} = \frac{{I_{in} D}}{{\left( {2 - D} \right)}}$$

The peak passing current of inductors *L*_1_ and *L*_2_ can be calculated according to relations (27) and (28).

27 $$I_{L1,pk} = \frac{{2I_{in} \left( {1 - D} \right)}}{{\left( {2 - D} \right)}} + \frac{{V_{in} \left( {1 - D} \right)}}{{2L_{1} f_{s} }}$$

28 $$I_{L2,pk} = \frac{{I_{in} D}}{{\left( {2 - D} \right)}} + \frac{{V_{in} D}}{{2L_{2} f_{s} }}$$

The RMS current passing through inductors *L*_1_ and *L*_2_ can be expressed by relations (29) and (30).

29 $$I_{L1,rms} = \sqrt {\left( {\frac{{2I_{in} \left( {1 - D} \right)}}{{\left( {2 - D} \right)}}} \right)^{2} + \left( {\frac{{V_{in} \left( {1 - D} \right)}}{{2\sqrt 3 L_{1} f_{s} }}} \right)^{2} }$$

30 $$I_{L2,rms} = \sqrt {\left( {\frac{{I_{in} D}}{{\left( {2 - D} \right)}}} \right)^{2} + \left( {\frac{{V_{in} D}}{{2\sqrt 3 L_{2} f_{s} }}} \right)^{2} }$$

#### Active switches selection

The voltage stress across switches *S*_1_ and *S*_2_ can be obtained using the relations (31) and (32).

31 $$V_{S1} = \frac{{V_{in} }}{D}$$

32 $$V_{S2} = \frac{{V_{in} }}{{\left( {1 - D} \right)}}$$

The average current passing through switches *S*_1_ and *S*_2_ is as follows:

33 $$I_{S1,avg} = \left( {1 - D} \right)I_{in}$$

34 $$I_{S2,avg} = DI_{in}$$

The peak current passing through switches *S*_1_ and *S*_2_ is obtained according to (35).

35 $$I_{S1,pk} = I_{S2,pk} = I_{in}$$

The root mean square (RMS) values of the switch's current can be obtained using the relations (31) and (32).

36 $$I_{S1,rms} = \sqrt {\left( {1 - D} \right)\left( {I_{in} } \right)^{2} }$$

37 $$I_{S2,rms} = \sqrt {D\left( {I_{in} } \right)^{2} }$$

#### Diode selection

The voltage stress across the diodes in the proposed structure can be calculated using the following equation:

38 $$V_{D} = \frac{{V_{in} }}{{D\left( {1 - D} \right)}}$$

The average current passing through all the diodes is as follows:

39 $$I_{D,avg} = I_{o}$$

The RMS current passing through diodes *D*_1_, *D*_2_, and *D*_3_ can be expressed by relation (40).

40 $$\left\{ {\begin{array}{*{20}c} {I_{D1,rms} = I_{D2,rms} = I_{o} \sqrt {\frac{1.12}{D}} } \\ {I_{D3,rms} = I_{o} \sqrt {\frac{1}{{\left( {1 - D} \right)}}} } \\ \end{array} } \right.$$

#### Capacitor selection

An important factor in choosing a capacitor is its permissible voltage ripple. Using Eq. (2) and the values of the average current of the inductor, the voltage ripple of the capacitors can be obtained as follows.

41 $$\left\{ {\begin{array}{*{20}c} {\Delta V_{C1} = \frac{{I_{o} }}{{C_{1} f_{s} }}} \\ {\Delta V_{C2} = \frac{{I_{o} }}{{C_{2} f_{s} }}} \\ {\Delta V_{C3} = \frac{{I_{o} D}}{{C_{3} f_{s} }}} \\ \end{array} } \right.$$

Allowable voltage ripple of capacitors is considered to achieve proper performance and low power loss. Using Eq. (41), the capacitor capacity can be derived as follows:

42 $$\left\{ {\begin{array}{*{20}c} {C_{1} = \frac{{I_{o} }}{{\Delta V_{C1} f_{s} }}} \\ {C_{2} = \frac{{I_{o} }}{{\Delta V_{C2} f_{s} }}} \\ {C_{3} = \frac{{I_{o} D}}{{\Delta V_{C3} f_{s} }}} \\ \end{array} } \right.$$

### Parameter design in operating region 2 (0.5 ≤ *D* < 1)

#### Inductor selection

In this operating region, considering that the converter has three operating modes, depending on which of the operating modes is selected and according to the equations governing the considered mode and voltage of capacitors, the current ripple of inductors can be expressed according to relations (43) and (44).

43 $$\Delta i_{L1} = \frac{{V_{in} D}}{{L_{1} f_{s} }}$$

44 $$\Delta i_{L2} = \frac{{V_{in} D}}{{L_{2} f_{s} }}$$

Hence, the values of inductors *L*_1_ and *L*_2_ operating under continuous conduction mode (CCM) are obtained according to relations (45) and (46).

45 $$L_{1} = \frac{{V_{in} D}}{{\Delta i_{L1} f_{s} }}$$

46 $$L_{2} = \frac{{V_{in} D}}{{\Delta i_{L2} f_{s} }}$$

The average current through the inductors *L*_1_ and *L*_2_ in operation region 2 is as follows:

47 $$I_{L1,avg} = \frac{{2I_{in} }}{3}$$

48 $$I_{L2,avg} = \frac{{I_{in} }}{3}$$

The peak current of inductors *L*_1_ and *L*_2_ can be calculated according to the relations (49) and (50).

49 $$I_{L1,pk} = \frac{{2I_{in} }}{3} + \frac{{V_{in} D}}{{2L_{1} f_{s} }}$$

50 $$I_{L2,pk} = \frac{{I_{in} }}{3} + \frac{{V_{in} D}}{{2L_{2} f_{s} }}$$

The RMS current of inductors *L*_1_ and *L*_2_ can be expressed by relations (51) and (52).

51 $$I_{L1,rms} = \sqrt {\left( {\frac{{2I_{in} }}{3}} \right)^{2} + \left( {\frac{{V_{in} D}}{{2\sqrt 3 L_{1} f_{s} }}} \right)^{2} }$$

52 $$I_{L2,rms} = \sqrt {\left( {\frac{{I_{in} }}{3}} \right)^{2} + \left( {\frac{{V_{in} D}}{{2\sqrt 3 L_{2} f_{s} }}} \right)^{2} }$$

#### Active switches selection

The voltage stress of switches *S*_1_ and *S*_2_ can be obtained using the following relation:

53 $$V_{S1} = V_{S2} = \frac{{V_{in} }}{{\left( {1 - D} \right)}}$$

The average current passing through switches *S*_1_ and *S*_2_ can be expressed according to Eq. (54) and their peak current can be expressed according to Eq. (55).

54 $$\left\{ {\begin{array}{*{20}c} {I_{S1,avg} = \frac{{I_{in} }}{3}\left( {1 + D} \right)} \\ {I_{S2,avg} = \frac{{I_{in} }}{3}\left( {2 - D} \right)} \\ \end{array} } \right.$$

55 $$I_{S1,pk} = I_{S2,pk} = I_{in}$$

#### Diode selection

The voltage stress of the diodes can be obtained using the Eq. (56) below.

56 $$V_{D} = \frac{{2V_{in} }}{{\left( {1 - D} \right)}}$$

The average current passing through diodes can be expressed according to Eq. (57) and the RMS current passing through diodes *D*_1_, *D*_2_, and *D*_3_ can be expressed by Eq. (58).

57 $$I_{D,avg} = I_{o}$$

58 $$I_{D1,rms} = I_{D2,rms} = I_{D3,rms} = I_{o} \sqrt {\frac{1}{{\left( {1 - D} \right)}}}$$

#### Capacitor selection

An important factor in choosing a capacitor is its permissible voltage ripple. Equation (59) can be used to determine the voltage ripple of the capacitors based on the average current values of the inductors and the equations governing each of the three considered modes. Equation (60) can be used to calculate the capacitor capacity of the proposed converter for operating region 2.

59 $$\left\{ {\begin{array}{*{20}c} {\Delta v_{C1} = \frac{{I_{o} }}{{C_{1} f_{s} }}} \\ {\Delta v_{C2} = \frac{{I_{o} }}{{C_{2} f_{s} }}} \\ {\Delta v_{C3} = \frac{{I_{o} }}{{C_{3} f_{s} }}D} \\ \end{array} } \right.$$

60 $$\left\{ {\begin{array}{*{20}c} {C_{1} = \frac{{I_{o} }}{{\Delta v_{C1} f_{s} }}} \\ {C_{2} = \frac{{I_{o} }}{{\Delta v_{C2} f_{s} }}} \\ {C_{3} = \frac{{I_{o} }}{{\Delta v_{C3} f_{s} }}D} \\ \end{array} } \right.$$

### Analysis of power loss and efficiency

As previously mentioned, parasitic resistances cause power losses and affect the performance and efficiency of converters. Different power losses in a converter must be considered and calculated. The power loss of a converter includes switch losses, which include conduction and switching losses, diode losses, inductor losses, and capacitor losses. The total power loss is obtained by summing all the described losses. In the following, each of the above losses is examined and how to calculate them is explained.

The switch losses are classified into two groups conductive and switching losses, which are calculated as relations (61) and (62), respectively.

61 $$P_{Loss}^{Switch} = P_{Conduction}^{Switch} + P_{Switching}^{Switch}$$

62 $$\left\{ \begin{gathered} P_{Conduction}^{Switch} = \left( {R_{{DS_{1} (on)}} I_{{S_{1} ,rms}}^{2} } \right) + \left( {R_{{DS_{2} (on)}} I_{{S_{2} ,rms}}^{2} } \right) \hfill \\ P_{Switching}^{Switch} = \left( {\frac{1}{2}V_{{DS_{1} (on)}} I_{{S_{1} ,avg}} \left( {t_{{r_{1} }} + t_{{f_{1} }} } \right)f_{s} } \right) + \left( {\frac{1}{2}V_{{DS_{2} (on)}} I_{{S_{2} ,avg}} \left( {t_{{r_{2} }} + t_{{f_{2} }} } \right)f_{s} } \right) \hfill \\ \end{gathered} \right.$$

where *V*_*DS*_ is the switch’s standing voltage and *I*_*S,avg*_ is the average current flowing through the switch. The switch’s rise and fall times are described as (*t*_*r*_, *t*_*f*_). The Switching frequency is *f*_*s*_ and finally *I*_*S,rms*_ indicate the effective current passing through the switch. The power losses of diodes can be calculated from the following equation, where *r*_*D*_ is the diode conduction resistance, *I*_*D,rms*_ is the effective current that flows through the diode, *V*_*D*_ is the diode's forward voltage drop, and *I*_*D,avg*_ is the average current that flows through the diode.

63 $$P_{Loss}^{Diode} = \left( {r_{{D_{1} }} I_{{D_{1} ,rms}}^{2} + V_{{D_{1} }} I_{{D_{1} ,avg}} } \right) + \left( {r_{{D_{2} }} I_{{D_{2} ,rms}}^{2} + V_{{D_{2} }} I_{{D_{2} ,avg}} } \right) + \left( {r_{{D_{3} }} I_{{D_{3} ,rms}}^{2} + V_{{D_{3} }} I_{{D_{3} ,avg}} } \right)$$

Similarly, relations (64) and (65) can be used to calculate the power losses of inductors and capacitors, respectively.

64 $$P_{Loss}^{Inductor} = r_{{L_{1} }} I_{{L_{1} ,rms}}^{2} + r_{{L_{2} }} I_{{L_{2} ,rms}}^{2} + r_{{L_{3} }} I_{{L_{3} ,rms}}^{2}$$

65 $$P_{Loss}^{Capacitor} = r_{{C_{1} }} I_{{C_{1} ,rms}}^{2} + r_{{C_{2} }} I_{{C_{2} ,rms}}^{2} + r_{{C_{3} }} I_{{C_{3} ,rms}}^{2} + r_{{C_{4} }} I_{{C_{4} ,rms}}^{2}$$

where *r*_*L*_ is the equivalent series resistance or ESR of the inductor, *I*_*L,rms*_ is the effective current that passes through the inductor, *r*_*C*_ is the equivalent series resistance or ESR of the capacitor, and *I*_*C,rms*_ is the effective current that passes through the capacitor.

With all of the aforementioned losses taken into account, relations (66) and (67) yield the total power losses and converter efficiency, respectively.

66 $$P_{Loss}^{Total} = P_{Loss}^{Switch} + P_{Loss}^{Diode} + P_{Loss}^{Inductor} + P_{Loss}^{Capacitor}$$

67 $$\eta = \frac{{P_{o} }}{{P_{o} + P_{Loss}^{Total} }} \times 100\%$$

**Figure 7:**
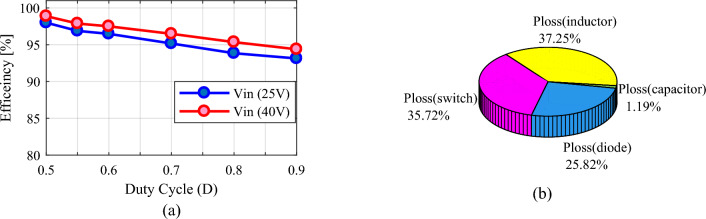
Analysis of efficiency and power losses of the proposed converter. (**a**) Efficiency according to duty cycle at constant power of 200 W and different input voltage. (**b**) Power loss distribution.

To analyze the losses and obtain the efficiency of the proposed converter, the parasitic values are considered according to Table 1. Figure 7 shows the efficiency and power loss analysis of the proposed converter in different duty cycles. The efficiency according to the duty cycle at a constant power of 200 W for input voltages of 25 V and 40 V is represented in Fig. 7a. According to this figure, for a higher input voltage and at a given power, the current passing through the elements is lower, and better efficiency is obtained. According to this figure, the efficiency of the proposed converter in a wide range of duty cycles and different input voltages is suitable and acceptable and provides values of 93%-98%. Furthermore, Fig. 7b displays the power loss distribution of the proposed converter at* D* = 0.55 and *V*_*in*_ = 25 V. The power loss distribution is determined by the proposed converter in MATLAB software using equations described in relations (61)–(67) for an output power of 200 W. According to Eq. (61), the losses caused by switches are equal to 2.2934 W. Diode losses using Eq. (63) are obtained at 1.658 W. The losses resulting from inductors and capacitors are 2.3915 W and 0.07657 W, respectively, according to Eqs. (64) and (65). Finally, the converter efficiency is 96.89% and the total losses, including the sum of the aforementioned losses, are 6.419 W.

**Table 1:** The parasitic values of the proposed converter for loss analysis and real gain factor.

Parasitic parameter	Values
Switches’ on-state resistance	r_DS_ = 0.04 Ω
Diodes’ on-state resistance	r_d_ = 0.17 Ω
Diodes’ forward voltage drop	V_FD_ = 0.7 V
Equivalent resistance of the capacitors	r_C_ = 10 mΩ
Equivalent resistance of the inductors	r_L_ = 100 mΩ

## Comparison of interleaved converters

**Figure 8:**
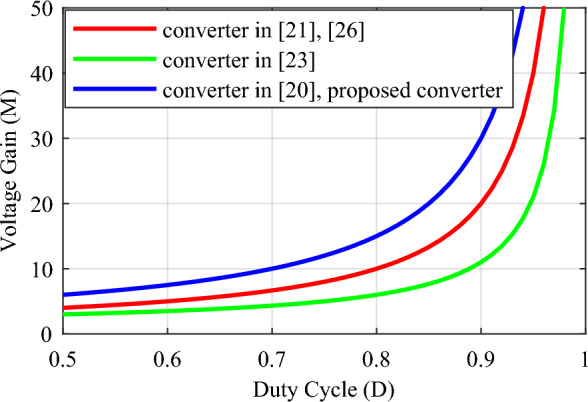
Voltage gain according to the duty cycle (*D*).

**Figure 9:**
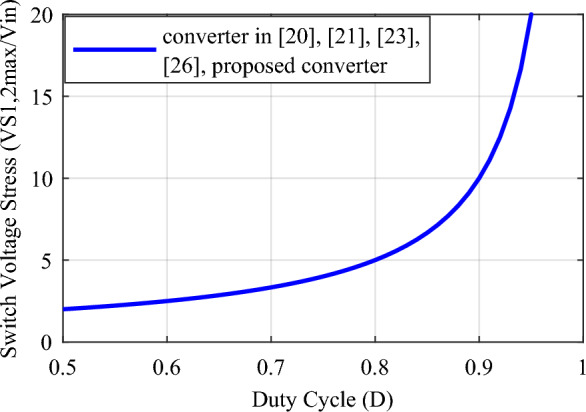
Switch's voltage stress according to the duty cycle (*D*).

**Figure 10:**
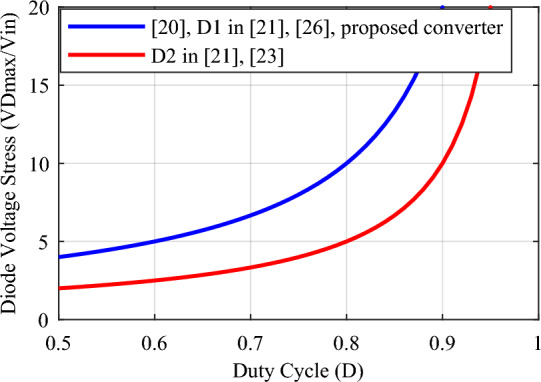
Voltage stress on the diodes according to the duty cycle (*D*).

A comparison with some similar converters described in^20,21,23^, and^26^ under the same conditions has been conducted to validate the proposed converter. and for the duty cycle of 0.5 ≤ *D* < 1. Table 2 presents the results of the comparison of these converters with the proposed converter according to the number of elements, voltage gain, maximum voltage stress of switches, maximum voltage stress of diodes, efficiency, continuous input current, and common ground. According to the total number of elements, the conditions for the structures under comparison are close to each other and the total number of elements in the structures of^21,23^, and^26^ is 8, 11, and 9 elements, respectively, and it is equal to 10 in the proposed converter and other structure. The relationship of voltage gain (*M*) according to duty cycle (*D*) for different structures is presented in Table 2 and Fig. 8 shows the structures’ voltage gain. According to this figure, the proposed converter's voltage gain for the same duty cycle is better than the structures under comparison. Also, the voltage gain of the proposed converter is even better than the structure^23^, which has more elements. Table 2 presents the maximum voltage stress relationship on the switches according to the duty cycle (*D*), and Fig. 9 illustrates the maximum voltage stress of the switches in the comparison structures. This figure illustrates that, although the proposed converter has a higher ideal voltage gain, the voltage stress on the switches is consistent across all compared structures, and they are all positioned similarly. In Fig. 10, the maximum voltage stress on the diodes is also displayed according to the duty cycle (*D*). Considering that the voltage gain of the proposed converter is greater than that of other structures, it is natural that the maximum voltage stress of the diodes in the proposed converter is higher than some comparative structures. In addition, according to Fig. 10, in the structure of^20,26^, and diode *D*_1_ of^21^, the voltage stress governing the diodes is the same as the proposed converter. In Table 2, the efficiency of the compared and proposed structures is presented. It is worth noting that the proposed converter demonstrates higher efficiency compared to the other structures. While the structure^23^ is reported to have an efficiency of 98.6% at a power of 900 W, this value is reported without considering the same conditions as the proposed converter. Based on Fig. 12, when the same conditions as those in Table 1 for the proposed converter are applied to this structure within the same power range, it is evident that the proposed converter outperforms it and exhibits superior efficiency.

**Table 2:** Comparison of the characteristics of the proposed converter and some references.

	Converter in^20^	Converter in^21^	Converter in^23^	Converter in^26^	Proposed converter
Number of switches	2	2	2	2	2
Number of inductors	2	2	2	2	2
Number of capacitors	3	2	3	3	3
Number of diodes	3	2	4	2	3
Total number of components	10	8	11	9	10
$M = \frac{{V_{o} }}{{V_{in} }}$	$\frac{ - 3}{{\left( {1 - D} \right)}}$	$\frac{2}{{\left( {1 - D} \right)}}$	$\frac{{\left( {2 - D} \right)}}{{\left( {1 - D} \right)}}$	$\frac{2}{{\left( {1 - D} \right)}}$	$\frac{3}{{\left( {1 - D} \right)}}$
$\frac{{V_{S1,2\max } }}{{V_{in} }}$	$\frac{1}{{\left( {1 - D} \right)}}$	$\frac{1}{{\left( {1 - D} \right)}}$	$\frac{1}{{\left( {1 - D} \right)}}$	$\frac{1}{{\left( {1 - D} \right)}}$	$\frac{1}{{\left( {1 - D} \right)}}$
$\frac{{V_{D,\max } }}{{V_{in} }}$	$$\left\{ {\begin{array}{*{20}c} {\frac{{V_{D1,\max } }}{{V_{in} }} = \frac{2}{{\left( {1 - D} \right)}}} \\ {\frac{{V_{D2,\max } }}{{V_{in} }} = \frac{2}{{\left( {1 - D} \right)}}} \\ {\frac{{V_{D3,\max } }}{{V_{in} }} = \frac{2}{{\left( {1 - D} \right)}}} \\ \end{array} } \right.$$	$$\left\{ {\begin{array}{*{20}c} {\frac{{V_{D1,\max } }}{{V_{in} }} = \frac{2}{{\left( {1 - D} \right)}}} \\ {\frac{{V_{D2,\max } }}{{V_{in} }} = \frac{1}{{\left( {1 - D} \right)}}} \\ \end{array} } \right.$$	$$\left\{ {\begin{array}{*{20}c} {\frac{{V_{D1,\max } }}{{V_{in} }} = \frac{1}{{\left( {1 - D} \right)}}} \\ {\frac{{V_{D2,\max } }}{{V_{in} }} = \frac{1}{{\left( {1 - D} \right)}}} \\ {\frac{{V_{D3,\max } }}{{V_{in} }} = \frac{1}{{\left( {1 - D} \right)}}} \\ {\frac{{V_{D4,\max } }}{{V_{in} }} = \frac{1}{{\left( {1 - D} \right)}}} \\ \end{array} } \right.$$	$$\left\{ {\begin{array}{*{20}c} {\frac{{V_{D1,\max } }}{{V_{in} }} = \frac{2}{{\left( {1 - D} \right)}}} \\ {\frac{{V_{D2,\max } }}{{V_{in} }} = \frac{2}{{\left( {1 - D} \right)}}} \\ \end{array} } \right.$$	$$\left\{ {\begin{array}{*{20}c} {\frac{{V_{D1,\max } }}{{V_{in} }} = \frac{2}{{\left( {1 - D} \right)}}} \\ {\frac{{V_{D2,\max } }}{{V_{in} }} = \frac{2}{{\left( {1 - D} \right)}}} \\ {\frac{{V_{D3,\max } }}{{V_{in} }} = \frac{2}{{\left( {1 - D} \right)}}} \\ \end{array} } \right.$$
Reported efficiency	93.56% (P_o_ = 300 W)	91.24% (P_o_ = 62 W)	98.6% (P_o_ = 900 W)	93.76% (P_o_ = 200 W)	96.89% (P_o_ = 200 W)
Continuous input current	Yes	Yes	Yes	Yes	Yes
Common ground	No	Yes	Yes	No	No

**Figure 11:**
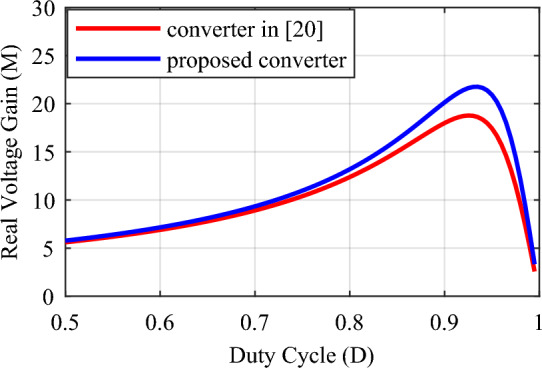
The parasitic voltage gain of the proposed converter and structure^20^.

The real voltage gain of the proposed converter and the structure^20^ are plotted in Fig. 11 for the same parasitic values for both converters and according to the duty cycle (*D*). Although the number of components, ideal voltage gain, voltage stress of the switch and diode, and other parameters of the structure^20^ are identical to those of the proposed structure, in practical conditions and terms of parasitic resistances, its real voltage gain is lower than the proposed converter for duty cycle in the range of 0.8 ≤ *D* < 0.95. This shows the relative superiority of the proposed converter over this structure. Also, the polarity of the output voltage in this structure is reversed, which causes a limitation in its performance, which does not exist in the proposed converter.

**Figure 12:**
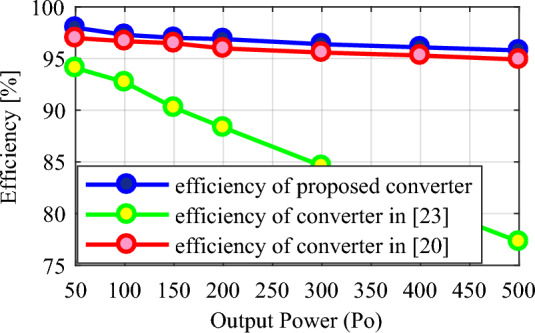
Efficiency of the proposed converter and structures^20^ and^23^.

Figure 12 shows the efficiency of the proposed converter and structures^20^ and^23^ considering the same parasitic values. According to this figure, the efficiency of the proposed converter is better than these two structures. The structure^23^ has more elements with a lower voltage gain factor, which causes higher losses and affects the efficiency of this converter. Finally, the proposed converter can be considered a better option compared to the mentioned references, considering that it has relative superiority in comparison.

## Simulation and experimental results

### Simulation results

**Figure 13:**
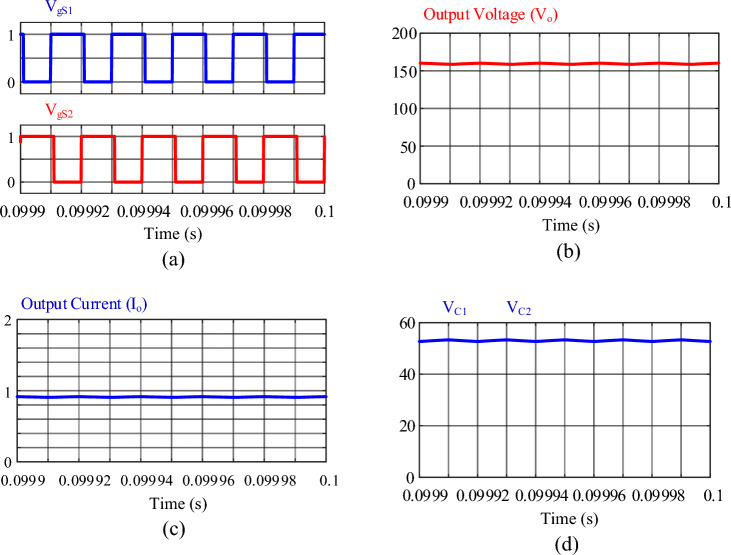
Results of the simulation: (**a**) switch gate signals (*V*_*gS*1_, *V*_*gS*2_), (**b**) output voltage (*V*_*o*_), (**c**) output current (*I*_*o*_), (**d**) voltage of capacitors *C*_1_ and *C*_2_.

**Figure 14:**
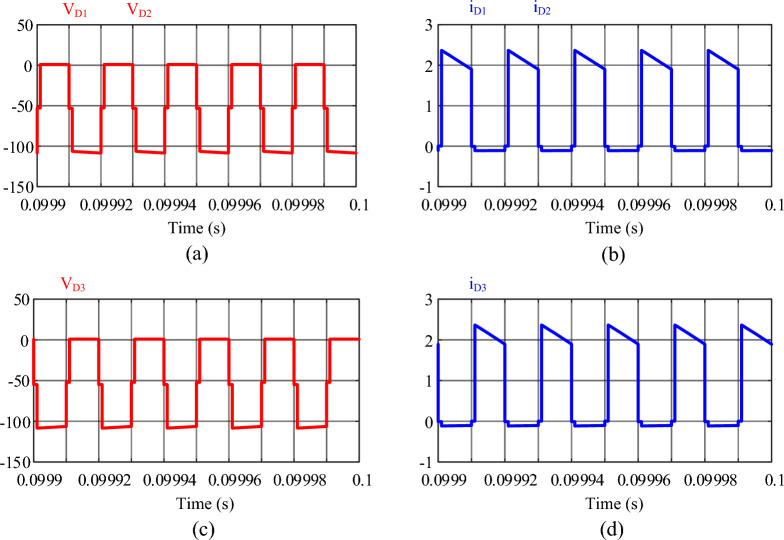
Results of the simulation: (**a**) voltage of diodes *D*_1_ and *D*_2_, (**b**) current of diodes *D*_1_ and *D*_2_, (**c**) voltage of diode *D*_3_, (**d**) current of diode *D*_3_.

**Figure 15:**
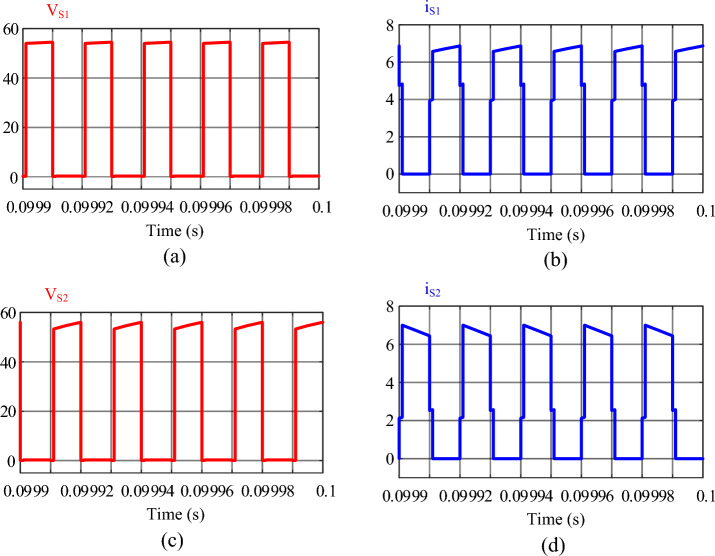
Results of the simulation: (**a**) voltage of switch *S*_1_, (**b**) current of switch *S*_1_, (**c**) voltage of switch *S*_2_, (**d**) current of switch *S*_2_.

**Figure 16:**
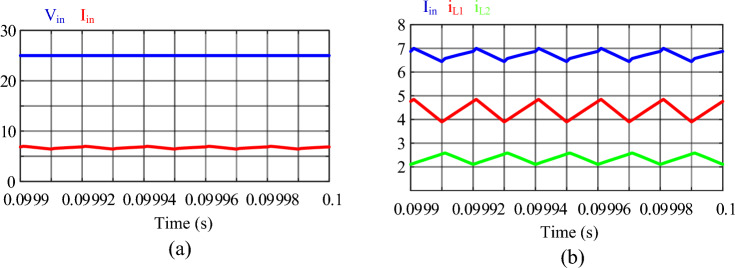
Results of the simulation: (**a**) input voltage and current, (**b**) input current, current of inductor *L*_1_ and current of inductor *L*_2_.

The MATLAB software simulations have been done with the parameters listed in Table 3 to verify the proposed converter’s operation. The results of the simulation are displayed in Figs. 13, 14, 15 and 16. It is worth noting that to simulate the real conditions, the simulation has been done considering the parasitic parameters and for the operating region 2 (0.5 ≤ *D* < 1). The values of the parasitic parameters have been applied in the simulation according to the information in Table 1.

**Table 3:** Parameters of the proposed converter.

Parameter	Values
Input voltage (*V*_*in*_)	25 V
Output voltage (*V*_*o*_)	159.5 V
Switching frequency (*f*_*s*_)	50 kHz
Output Load	157 Ω
Duty Cycle (*D*)	0.55
Inductor L_1_	200 μH
Inductor L_2_	500 μH
Capacitor C_1_	47 μF
Capacitor C_2_	47 μF
Capacitor C_3_	10 μF
MOSFETS (*S*1, *S*2)	IRFP260N
Diodes (*D*_1_, *D*_2_, *D*_3_)	MBR20B200

The gate signals of switches *S*_1_ and *S*_2_ are shown in Fig. 13a, which work with a duty cycle of 0.55 and are switched at a 180^◦^ phase difference from each other. According to Fig. 13b, the output voltage is equal to 159.5 V. Considering the ideal and real voltage gain of the proposed converter in Eqs. (18) and (20) respectively, the output voltage of the converter is 166 V in the ideal state and 159.88 V in the real state, which confirms the results of the simulation. It should be noted that if a switch and diodes with lower on-state resistance are used, the difference between the ideal and real conditions is reduced. Figure 13c shows the output current of the converter; the average value of the output current is about 0.95 A. The voltage of capacitors *C*_1_ and *C*_2_ is shown in Fig. 13d, the average of which is 53.04 V. According to Eq. (17), the voltage of capacitors *C*_1_ and *C*_2_ is equal to 55.55 V, which is very close to the theoretical value.

Figure 14 shows the voltages and currents passing through diodes *D*_1_, *D*_2_, and *D*_3_. Because diodes *D*_1_ and *D*_2_ turn on and off in the same modes, they have the same voltage and current waveforms. Also, according to Eq. (56), all three diodes experience the same voltage. In Fig. 14a and c, this value is 108.5 V, which is close to the theoretical value of 111.1 V. In Fig. 14b and d the current of the diodes is shown, according to the Eq. (57), its average value is almost equal to the output current and about 0.95 A.

Figure 15 shows the voltage and current of switches *S*_1_ and *S*_2_, respectively. The voltage stress of the switches is 54.51 V, which is consistent with Eq. (53). Also, the voltage stress of the switches is much lower than the output voltage and about one-third of the output voltage, which is a good advantage for the converter. The current passing through switches *S*_1_ and *S*_2_ are shown in Fig. 15b and d, respectively. The peak current passing through the switches is 6.871 A.

In Fig. 16a the input voltage and current can be seen. As mentioned in Table 3, the input voltage is 25 V. The average value of the input current is equal to 6.728 A, which can be seen in Fig. 16b. Also, the average current of inductors *L*_1_ and *L*_2_ are 4.378 A and 2.350 A, respectively. It is worth mentioning that the input current ripple is less than the current ripple of inductors, which is the result of using the interleaved technique in the proposed converter.

### Experimental results

**Figure 17:**
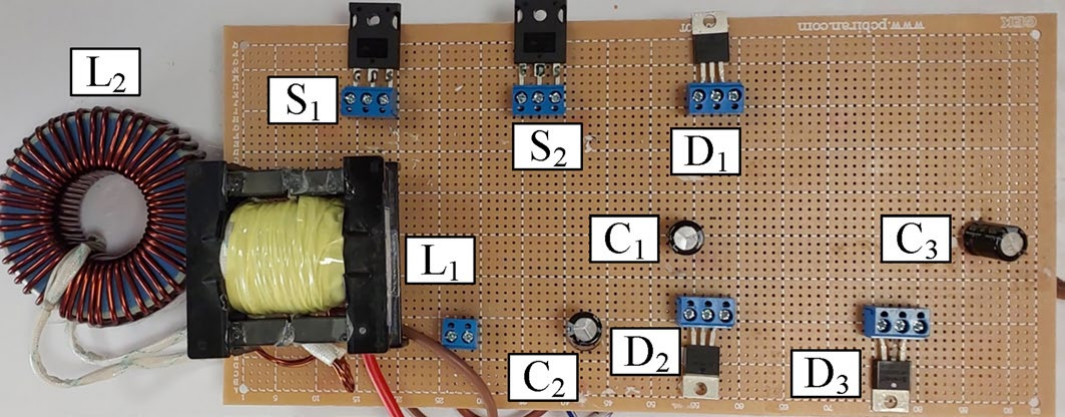
Experimental prototype.

**Figure 18:**
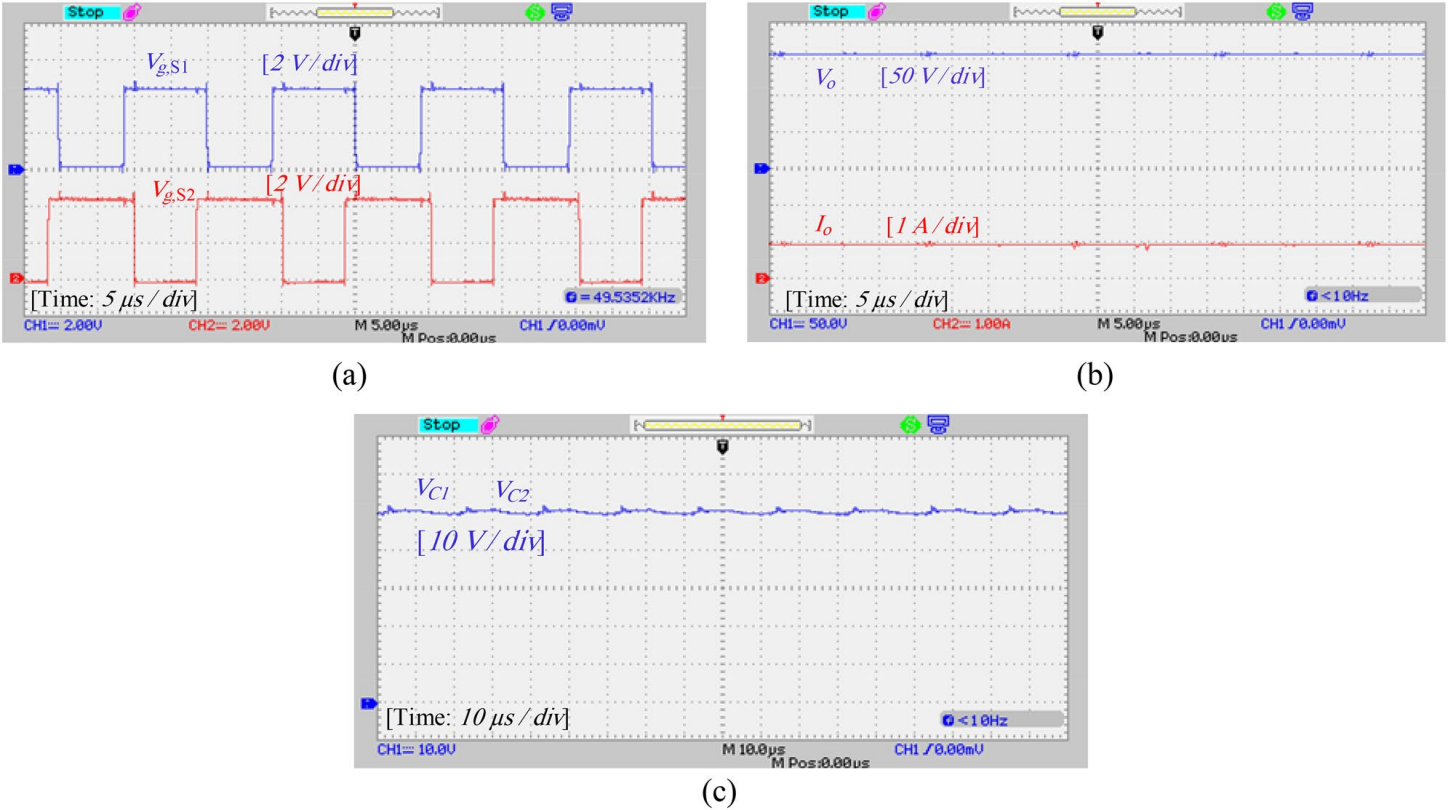
Experimental results: (**a**) gate signals of switches (*V*_*gS*1_, *V*_*gS*2_), (**b**) output voltage and output current (*V*_o_, *I*_o_), (**c**) voltage of capacitors *C*_1_ and *C*_2_.

**Figure 19:**
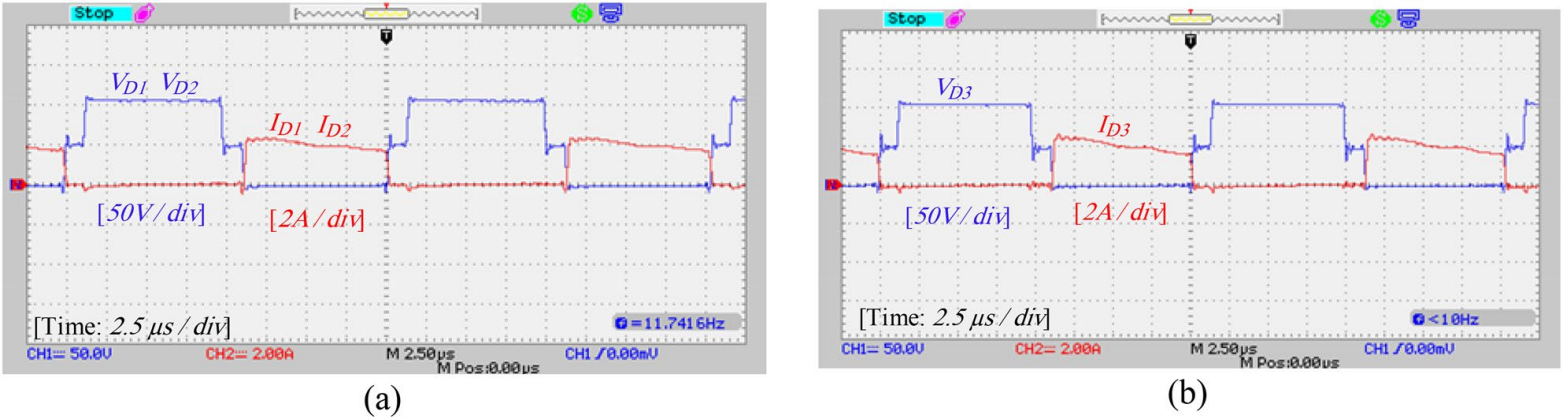
Experimental results: (**a**) voltage and current of diodes *D*_1_ and *D*_2_, (**b**) voltage and current of diode *D*_3_.

**Figure 20:**
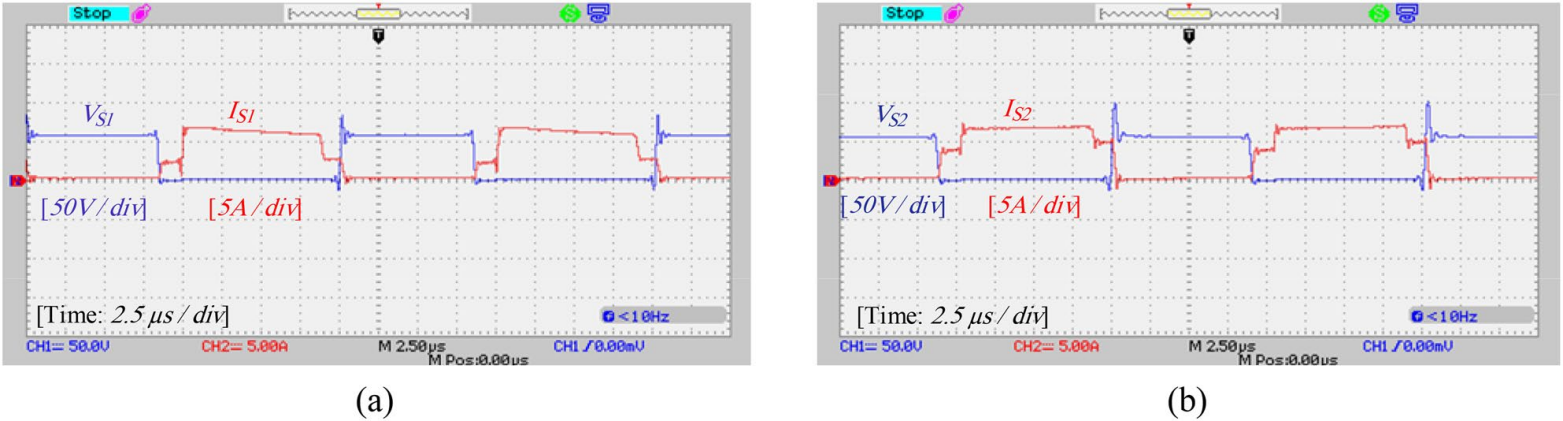
Experimental results: (**a**) voltage and current of switch *S*_1_, (**b**) voltage and current of switch *S*_2_.

**Figure 21:**
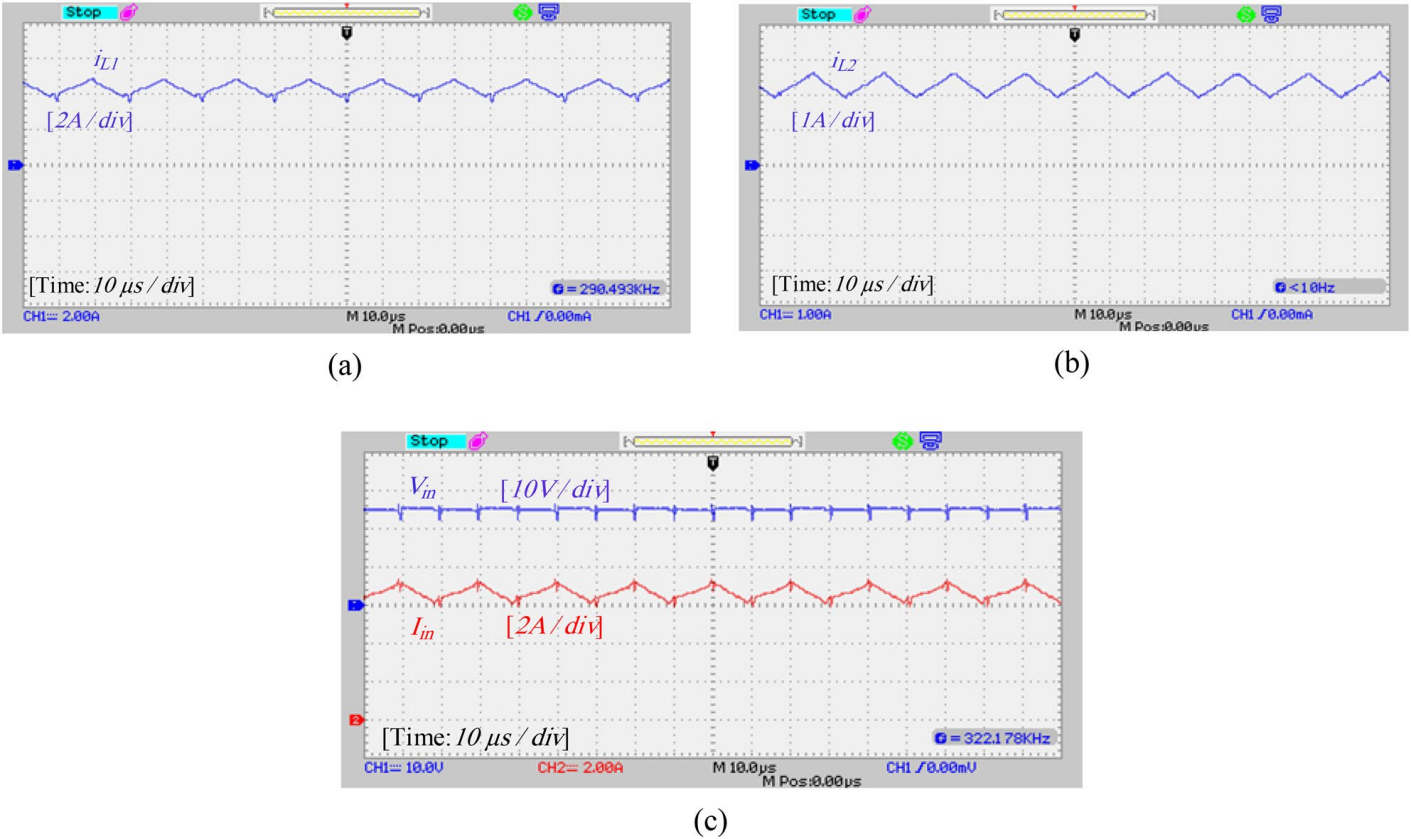
Experimental results: (**a**) current of inductor *L*_1_, (**b**) current of inductor *L*_2_, (**c**) input voltage and current.

A laboratory prototype designed to verify the proposed converter’s specifications, theoretical calculations, and simulation results is depicted in Fig. 17. The parameters in Table 3 have been used to set up the laboratory sample along with the IRFP260N MOSFET and MBR20B200 diode. The results and waveforms of the proposed converter in continuous conduction mode (CCM) and at a switching frequency of 50 kHz with *D* = 0.55 are shown in Figs. 18, 19, 20 and 21.

The gate signal of the switches is shown in Fig. 18a. As mentioned before, these signals have a phase difference of 180^◦^. Figure 18b shows the output voltage and current. According to the simulation results, the output voltage at a duty cycle of 55% and an input voltage of 25 V is approximately 159 V. Also, the average value of the output current is 0.94 A, which confirms the results of the simulation. The voltage of capacitors *C*_1_ and *C*_2_ is shown in Fig. 18c. Theoretically, the voltage of capacitors is calculated as 55.55 V according to Eq. (17), which is nearly the same as the experimental result. It is worth noting that the difference between simulation and experimental values is due to losses in laboratory conditions.

According to Fig. 19a, the voltage and average current of diodes *D*_1_ and *D*_2_ are equal to 111 V and 0.94 A, respectively. Also, according to Fig. 19b, the voltage across diode *D*_3_ is equal to 108 V, and the average current passing through diode *D*_3_ is about 0.94 A, and these values are consistent with the simulation results and theoretical calculations. Also, according to Fig. 19, the maximum current passing through the diodes is 2 A which is consistent with the simulation results. Figures 20a and b show the voltage and current stress on the switches. According to this figure, the voltage stress on the switches is about 54 V and the peak current passing through the switches is about 6.8 A, which does not contradict the results of the simulation and the resulting relationships. Figure 21 also shows the input voltage and current and the current of the inductors, which according to Fig. 16, the experimental results and the simulation results match.

## Conclusion

In this paper, an interleaved DC-DC step-up converter with improved voltage gain based on a voltage multiplier rectifier is presented. The proposed interleaved DC-DC step-up converter has two operating regions (0 < *D* ≤ 0.5, 0.5 ≤ *D* < 1). The configuration of the proposed converter has been investigated and its operating modes have been carried out under continuous conduction mode (CCM). Ideal and real voltage gain have been calculated and compared with similar structures. The proposed converter has been compared to similar converters in terms of component count, voltage gain, and voltage stress on switches and diodes. The results that have been presented indicate that, for the same duty cycle, the voltage gain of the proposed converter is higher and, in some situations, equal to that of similar converters. The voltage stress of the switches is much lower than the output voltage. Taking into account the proposed converter's continuous input current and easy control circuit, the proposed converter seems like a good choice for DC microgrid systems. The proposed converter’s input current ripple is lower than the current ripple of the inductors due to the use of the interleaved technique, which reduces the need for filters. By taking into account the parasitic parameters, the efficiency, and real voltage gain have been measured accurately, and at 150 W power, the efficiency is 97%. The comparisons show that the proposed converter has suitable conditions in terms of voltage gain in both ideal and parasitic conditions. Simulation analysis is provided by MATLAB software to validate the mathematical calculations. Also, the experimental results of the laboratory prototype show full agreement with the simulation results.

## Data Availability

All data generated and analyzed during the current study are available from the corresponding author upon reasonable request.
